# Targeted Gene Delivery Therapies for Cervical Cancer

**DOI:** 10.3390/cancers12051301

**Published:** 2020-05-21

**Authors:** Ángela Áyen, Yaiza Jiménez Martínez, Houria Boulaiz

**Affiliations:** 1 Department of Dermatology, San Cecilio Universitary Hospital, 18016 Granada, Spain; aayen@correo.ugr.es; 2 Department of Human Anatomy and Embryology, University of Granada, 18016 Granada, Spain; 3 Biopathology and Medicine Regenerative Institute (IBIMER), University of Granada, 18016 Granada, Spain; yaijmartinez@correo.ugr.es; 4 Biosanitary Institute of Granada (ibs.GRANADA), SAS-Universidad de Granada, 18016 Granada, Spain; 5 Excellence Research Unit “Modeling Nature” (MNat), University of Granada, 18016 Granada, Spain

**Keywords:** cervical cancer, targeted gene therapy, delivery systems, immunopotentiation, cervical cancer stem cells

## Abstract

Despite being largely preventable through early vaccination and screening strategies, cervical cancer is the most common type of gynecological malignancy worldwide and constitutes one of the leading causes of cancer deaths in women. Patients with advanced or recurrent disease have a very poor prognosis; hence, novel therapeutic modalities to improve clinical outcomes in cervical malignancy are needed. In this regard, targeted gene delivery therapy is presented as a promising approach, which leads to the development of multiple strategies focused on different aspects. These range from altered gene restoration, immune system potentiation, and oncolytic virotherapy to the use of nanotechnology and the design of improved and enhanced gene delivery systems, among others. In the present manuscript, we review the current progress made in targeted gene delivery therapy for cervical cancer, the advantages and drawbacks and their clinical application. At present, multiple targeted gene delivery systems have been reported with encouraging preclinical results. However, the translation to humans has not yet shown a significant clinical benefit due principally to the lack of efficient vectors. Real efforts are being made to develop new gene delivery systems, to improve tumor targeting and to minimize toxicity in normal tissues.

## 1. Introduction

Over 570,000 women developed cervical cancer worldwide in 2018, with this being the fourth most frequent malignancy in women, causing 311,365 annual deaths with approximately 85% occurring in underdeveloped or developing countries [1]. In the developing world, there is inadequate or non-existent screening, and the standard treatment included in the guidelines is often absent or deficient [2]. Almost all cases are caused by persistent human papillomavirus (HPV) infection, its presence considered necessary to cause cervical cancer. HPV16 and HPV18 are the two most carcinogenic HPV types. Infection persists in a small percentage of women, and in some cases the viral genome is integrated into the DNA of the cervical cell, resulting in a genetic dysregulation and development of premalignant lesions and cancer. Some factors can increase the risk of pre-cancer and cancer among those women who are infected: smoking, multiparity, long-term oral contraceptive use and co-infection with other sexually transmitted agents (type 2 herpes virus or the human immunodeficiency virus) [3].

Cervical cancer usually arises from the cervical transformation zone or squamocolumnar junction, a dynamic zone where stratified squamous epithelium replaces the glandular epithelium [3]. About 75% of cervical cancers are squamous cell cancers and initiate in the metaplastic epithelium at the ectocervix, while the other 20–25% are adenocarcinomas and arise in the glandular epithelium of the endocervical canal [2]. The development of cervical cancer is a complex multistep process, from oncogenic HPV infection, squamous cervical intraepithelial lesion (CIN), carcinoma in situ to expansion of carcinoma and invasion [4]. This is a process that lasts for years, which justifies the development of screening strategies as a guide to the management of cervical cancer. Vaccination, by non-infectious, virus-like particles, is a promising primary prevention strategy, but ineffective in pre-existing HPV infections. Currently, there are three HPV vaccines that provide protection against high-risk HPV types 16 and 18: the bivalent vaccine, the quadrivalent vaccine (HPV 6, 11, 16 and 18) and, finally, the nonavalent vaccine (HPV 6, 11, 16, 18, 31, 33, 45, 52 and 58). HPV vaccine is recommended by the Advisory Committee on Immunization Practices at the age of 11 or 12 years, but can be given at up to 26 years of age. It is recommended that women follow cervical cancer screening procedures since not all oncogenic viruses are covered [5].

### 1.1. Diagnosis and Early Detection

Early detection and treatment of precancerous lesions can prevent most cervical cancers, and the use of screening methods in developed countries has meant a reduction in the incidences of mortality. Cytology (Papanicolau test or liquid-based cytology), HPV testing (more sensitive and negative predictive value but less specific than cytology methods), and visual inspection with acetic acid are the recommended screening tests. However, the optimal testing method and frequency are under debate, because the field of cervical cancer prevention is highly dynamic and clinical guidelines are modified as new evidence emerges [6]. When women have a pre-cancerous lesion or a positive screening test in a screen-and-treat approach, they must receive an appropriate treatment with cryotherapy, loop electrosurgical excision procedure (LEEP) or cold knife conization (CKC) [7].

**Figure 1:**
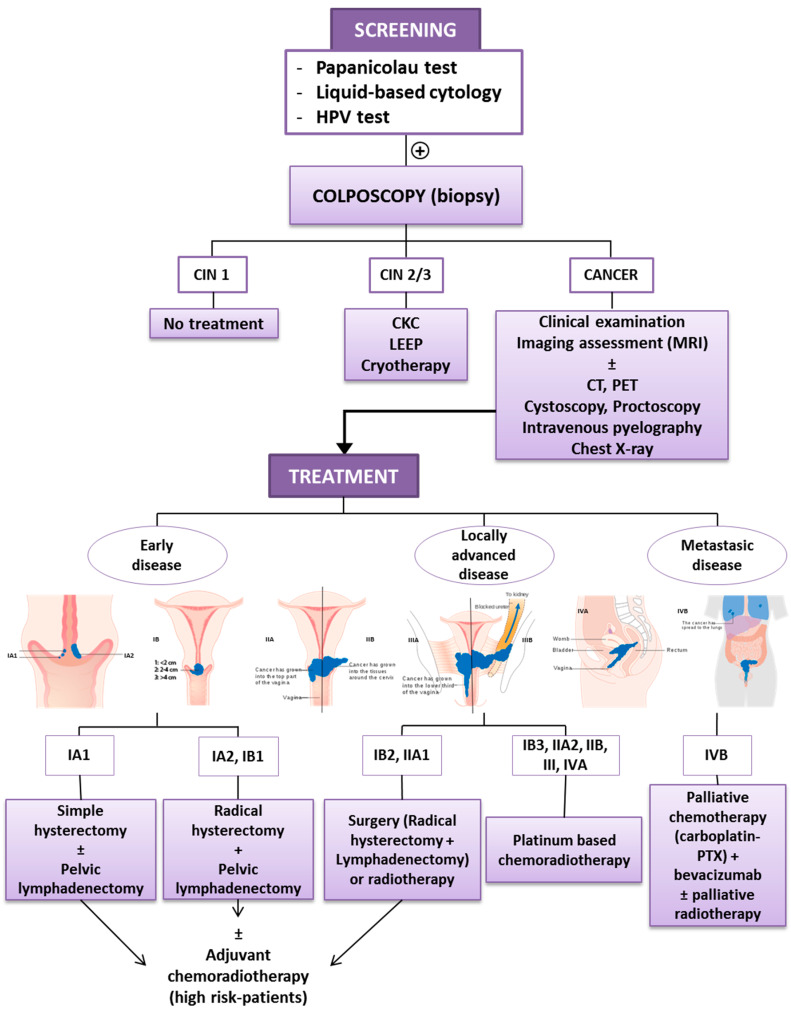
Algorithm for screening and treatment of cervical cancer. Cervical cancer stage images modified from Wikimedia Commons.

The standard management of patients with cervical cancer is based on the International Federation of Gynecology and Obstetrics (FIGO) stage, a staging system based on clinical examination (Figure 1). The recent revision by the FIGO Gynecologic Oncology Committee is based on imaging and pathological assessment of the pelvis and evaluation of pelvic and paraaortic lymph nodes: ultrasound, computed tomography (CT), magnetic resonance imaging (MRI) and positron emission tomography (PET). In selected patients, the use of other complementary tests (e.g., cystoscopy, proctoscopy, intravenous pyelography, ultrasound of the renal tract, and X-ray examination of the lungs and skeleton) helps in the evaluation of the extension of the disease. The diagnosis is based on the histopathological examination of removed tissue [8].

### 1.2. Current Treatment of Cervical Cancer

Women with early-stage disease (cancer limited to the uterus) have different treatment options: conization or trachelectomy if the patient wishes to preserve fertility and simple or radical hysterectomy in the other cases, along with pelvic lymphadenectomy in the case of lymphovascular space invasion or stage IA2 cancer. Radiotherapy (RT) is used for women with early stage diseases who are not candidates for surgery, with or without chemotherapy (depending on the risk factors). In tumors treated with surgery with unfavorable prognostic factors, adjuvant RT can be used, again with or without chemotherapy [8,9].

Women with locally advanced cervical cancer (stage IB2 to IVA) have a higher rate of recurrence and worse survival rates. In Stage IB2 and IIA1, surgery and RT have similar outcomes, since the standard management for Stage IB3 and IIA2 is platinum-based chemoradiotherapy (CRT) (weekly cisplatin for 5–6 cycles during the course of external beam RT and intracavitary brachytherapy). In the management of distant metastatic disease, doctors use palliative chemotherapy, carboplatin and paclitaxel (PTX) combination to reduce symptoms and improve quality of life, in combination with bevacizumab, recently approved for the treatment of metastatic or recurrent cervical cancer, and palliative radiotherapy [8,9]. Figure 1 shows a schematic representation of cervical cancer detection and the treatment procedure.

### 1.3. New Approaches for the Treatment of Advanced Cervical Cancer

Advances in molecular biology have led to the development of new targeted agents, including drugs targeting defective DNA repair, such as PARP inhibitors; drugs targeting angiogenesis; and immune check-point inhibitors such as anti-PD-1/PD-L1 antibodies [10]. Several clinical trials are currently underway, focusing on these therapeutic strategies for the treatment of cervical cancer. Immune checkpoint inhibitors, in particular the programmed death-1(PD-1)/PD-1 ligand (PD-L1) inhibitor, have been found to be useful in treating some types of cancer. The results of KEYNOTE-158, a phase II trial, led to the approval of pembrolizumab, a PD1-blocking antibody, for the treatment of advanced and recurrent cervical cancer expressing PD-L1, by the Food and Drug Administration (FDA) in the United States [11]. Clinical trial CheckMate 358 investigated nivolumab in a similar population, with an overall response rate of 26.3%, with a disease control rate of 70.8% [12]. Phase III trials investigating immune checkpoint inhibitors in cervical cancer are currently ongoing, such as EMPOWER-CERVICAL 1 which is comparing the anti-PD-1 agent cemiplimab with chemotherapy (NCT03257267). In addition, ongoing clinical studies in cervical cancer consist of combination therapy with radiation and/or chemotherapy, aiming to achieve a better response (NCT02635360, NCT03612791, and NCT01711515). For example, CALLA (NCT03830866) is a 714-patient clinical trial among women with locally advanced cervical cancer, which compares efficacy and safety of durvalumab with, and following, concurrent CRT versus concurrent CRT alone [13].

Bevacizumab is an anti-angiogenic agent currently used to treat advanced cervical cancer. The use of anti-angiogenic drugs can improve efficient tumor infiltration by immune cells, showing that combinations of anti-angiogenic agents with immune checkpoint inhibitors have potential antitumor effects. Several ongoing clinical trials have the aim of studying this potential effect alone (NCT02921269) or in combination with chemotherapy (NTC03912415, NCT03635567). Tyrosine Kinase Inhibitors (TKIs) with anti-angiogenic activity, such as cediranib, nintedanib, sunitinib and pazopanib, have been studied in advanced cervical cancer. The latter two have showed a poor response in their respective studies [14,15]. However, CIRCCa trials report significantly improved progression free survival (PFS) with six cycles of carboplatin–PTX plus daily cediranib [16]. A phase II study of nintedanib in patients with advanced or recurrent cervical cancer is currently underway (NCT02009579). 

The poly (ADP-ribose) polymerase (PARP) family of enzymes is involved in DNA repair pathways. Rucaparib, Niraparib, and Olaparib, molecules that inhibit these enzymes, have been approved by the FDA and the European Medicine Agency for the treatment of recurrent ovarian cancer. There are not yet any data that might confirm the potential efficacy of these drugs in advanced cervical cancer. Their role in the treatment of cervical cancer is currently being investigated, and there are ongoing trials studying the effect of veliparib, rucaparib or niraparib in advanced cervical cancer (NCT03795272, NCT03644342, and NCT03476798).

Despite all the efforts being made, the prognosis for advanced cervical disease remains poor [17], with a 5-year PFS rate of 56% in Stage III and 17% in metastatic disease. This has underlined the need to improve current standard care, driving the exploration for new treatments. Research advances in the understanding of the molecular biology of HPV-related tumors have identified several molecular targets for therapy, which present gene therapy as a promising strategy to attack these targets, and this is being studied in other gynecological cancers [18]. Developing this type of approach is bringing us closer to more precise, personalized cancer medicine with fewer associated side effects, transforming the management of cancer in general and cervical cancer in particular. In this review, we discuss targeted gene delivery therapy as a promising approach for cervical malignancies, paying special attention to the strategies developed to date and to clinical trials.

## 2. What’s New in Gene Targeted Therapies for Cervical Cancer?

Over the last few years, several novel strategies have been designed with the aim of targeting cervical cancer. The success of gene therapy depends on the development of safe gene vectors and gene delivery strategies that allow targeted cancer cells and minimize the off-target side effects that appear throughout the treatment [19,20]. However, despite the effort being made, gene-targeted therapy still has severe problems in terms of a drug delivery system, such as short half-life of the therapeutic gene, its easy degradation in plasma, a lack of efficient and nonspecific delivery of the gene due to the complexity in an in vivo system, inappropriate target identification, the acute immune response that could be caused, or high cost and difficulty relating to its preparation [21]. Moreover, intratumoral injection is problematic when treating recurrent or advanced cervical cancer tumors. Next, we will review different strategies aimed at targeting cervical cancer and the strategies followed to address their limitations.

### 2.1. Vectors Targeting Cervical Cancer

Vectors are the delivery systems used to introduce exogenous genes into the target cells. A wide variety of vectors have been used in experimental studies, but we can group them into two types: viral and non-viral systems [22]. The common viruses used as viral vectors are retrovirus, lentivirus, adenovirus (Ad), adeno associated virus (AAV) and simple herpes virus [21]. Non-viral vectors can be physical methods (such as electroporation, gene gun or ultrasound), or different types of smart molecules (liposomes, polyplexes, nanoparticles, etc.) able to enhance the therapeutic effect of current standard treatment modalities, including chemotherapies and radiotherapies [23,24]. Over time we have seen that there is no ideal delivery system, each has its advantages and disadvantages. Viral vectors usually have higher transfection efficiency due to their fusogenic cell receptor binding properties. However, their implementation into clinical therapy has been plagued with significant drawbacks. Conversely, non-viral vectors are characterized by their low immunogenicity and relative safety, but their transfection efficiency is lower than that of viral vectors [21,24].

The construction of vectors directed more specifically at the cancer cells through different forms of targeting produces improved results in gene therapy [25]. There are multiple ways to target cervical cancer cells, many based on molecules that are specifically expressed on their surfaces [26], others on more novel strategies, such as the use of stem cells [27], presenting interesting results in preclinical studies.

Folate receptor α (FRα) is a membrane-bound protein which mediates folate uptake by endocytosis, essential for DNA synthesis and, consequently, for replication, cell division and growth, especially in dividing cells [28]. These characteristics confer signaling and growth advantages to malignant cells. In addition, compared to normal tissues and cells, there was a higher expression in various epithelial tumors, including cervical cancer [29], and in endocervical adenocarcinomas [30]. This presents an interesting option of targeted delivery for gene therapy in cervical cancer, thereby reducing toxic side effects in healthy tissues. This feature was exploited in a study which developed FRα targeted nano-liposomes (FLP) to deliver a pigment epithelium-derived factor (*PEDF*) gene to HeLa cells showing high transfection efficiency and effective anti-tumor activity, not only of the *PEDF* gene but also of the vector itself [26]. Liu et al. developed polyethylene glycol-polylactic acid (PEG-PLA) nanocarriers and folate was linked onto these nanoparticles for targeting cancer cells through the FRα. The resulting gene loaded polymeric nanoparticles enhanced gene transfection efficiency (20% higher than DNA nanoparticles and 40% higher than naked DNA) and decreased cytotoxicity [31]. However, the main limitation of the use of FRα is its heterogeneous expression in cancer cells since not all cervical cancer patients express it. Wu et al. reported a lack of significant receptor expression quantitated in squamous carcinomas [29], and in another study among 25 woman with advanced cervical cancer, 9 exhibited negative FR expression and 16 exhibited positive expression [32]. Therefore, for its application in humans, it would be interesting to carry out a pre-screening study to determine tumor FRα expression and whether patients can benefit from this type of treatment.

Another strategy in targeting cervical cancer cells is the use of HPV E2 protein, whose functions are mediated through its binding to a palindromic sequence, E2 binding sites (E2BS), a promoter responsible for regulation of other HPV protein expression. Therefore, an E2-specific promotor could be used as a sequence to induce the expression of therapeutic genes selectively in HPV-infected cells, since this expression will only be induced in the presence of E2. In fact, Bermúdez-Morales et al. used E2BS to construct an HPV-specific promoter to drive the expression of interleukin (IL)-12, using an adenoviral vector, and showed it to be functional in vitro and in vivo [33]. However, disruption of this sequence during the chromosomal integration of HPV occurs frequently, thus limiting the scope of the vector and therefore lowering the effectiveness of the treatment.

Another system has been used by, Zhang et al., who have constructed fusion protein combining the genes for a human anti-EGFR single-chain antibody (*scFC*) and a truncated protamine (*tP*) with nucleic acid binding activity. Via this vector, they effectively delivered a small interfering RNA (siRNA) of the human wings apart-like gene (*hWAPL*) into cervical cancer HeLa cells, led by the antibody. This shows that this vector could be an effective carrier for targeted gene therapy and siRNA therapy of EGFR-positive cervical cancers. Nevertheless, although these results are encouraging, they still need to be validated in vivo before they can really be considered a possible therapeutic tool for use in humans [34]. Finally, genetically engineered stem cells (GESTECs) represent a potential vehicle to target gene delivery due to their ability to migrate towards cancer cells. Kim et al. found that the GESTECs selectively migrated to HeLa cervical cancer cells, via their tumor tropism derived from a response to several chemoattractant factors. These factors were secreted by cervical cancer cells and the action of related receptors produced by stem cells, mainly vascular endothelial growth factor (VEGF) and VEGF receptor (VEGFR)-2 [27]. The modification of stem cells to enhance their innate abilities such as tumor tropism and to confer them with new functionalities, together with their use for gene delivery, makes GESTECs a promising new tool for anticancer therapy.

The construction of vectors directed more specifically to the cancer cells through different forms of targeting allows us to improve the results of gene therapy. There are multiple ways to target cancer cells, many based on molecules that express themselves specifically on their surfaces, others on more novel strategies, such as the use of stem cells, all presenting interesting results in experimental studies. Nonetheless, and taking into account the limitations of the new systems tested, we believe that the probable best option would be to manufacture vectors targeting various molecular targets in order to increase their possibility of selective transfection and, hence, their bio-efficacy.

### 2.2. Targeted Therapy Strategies for Cervical Cancer Treatment

Diverse treatment approaches have been studied as selective gene therapies for cervical cancer; next, we will review the most relevant.

#### 2.2.1. Therapy Targeting Compensation of Mutations

Given that most mutations leading to cervical cancer development affect tumor suppressor genes, oncogenes, and DNA repair genes, the restoration of their normal function may be a good strategy (Table 1).

Prolonged infections of uterine cervix epithelium with HPV have been recognized as the main cause of the complex molecular changes leading to transformation of cervical epithelial cells. HPV is the most common sexually transmitted disease worldwide. HPV-16 and -18 are the most prevalent high-risk HPV types and account for approximately 63% and 16% of invasive cervical cancers respectively [10]. HPVs infect cells of the basal layer of stratified squamous epithelia, leaving HPV genomes as extrachromosomal elements. E1 and E2 are the first viral proteins to be expressed, and they regulate viral genome replication at a low copy number and transcription of viral proteins. After cell division, one daughter cell differentiates to form the epithelial suprabasal layer. The expression of E6 and E7 viral proteins deregulates cell cycle control, allowing these differentiated cells to enter the S phase, increasing viral genome copies [35]. Finally, virions get released with the epithelial desquamation, causing new cell infections. The E2 gene is responsible for repression of E6 and E7, as part of a mechanism to regulate the copy number. However, integration of viral DNA usually disrupts the E2 gene site, leading to the deregulated expression of viral genes, including E6 and E7, a crucial step in the progression to cancer [36].

E7 proteins target p105, better known as retinoblastoma protein (pRb), p107 and p130 for degradation. As a consequence, constitutive activation of E2F modulated gene expression programs occurs, which controls DNA synthesis and cell proliferation. In addition, E7 avoids G1 arrest during epithelial cell differentiation. E6 proteins inactivate the p53 tumor suppressor by targeting it for degradation through the 26S proteasome, abrogating its antiproliferative and proapoptotic functions. E6 expression also activates telomerase expression, allowing the maintenance of telomere integrity despite repeated cell divisions, and modulates activities of PDZ domain-containing proteins and tumor necrosis factor (TNF) receptors. The high-risk E5 protein co-operates with E6 and E7 to promote hyperproliferation of infected cells. E5 expression induces aberrant cellular proliferation through increased epidermal growth factor receptor (EGFR) signaling and activation of the MAPK pathway. This expression also inhibits apoptosis through the degradation of Fas receptors, prevents the formation of a death domain, and helps to prevent the attack of infected cells by the immune system [36].

For all of the above, the restoration of genes altered by HPV infection has been exploited by several researchers to design new gene therapy strategies targeting cervical cancer.

##### Tumor Suppressor Gene Restoration

There are multiple tumor suppressor genes, whose restoration has been studied in cervical cancer cells, with acceptable results in terms of growth inhibition and apoptosis effects.

**Figure 2:**
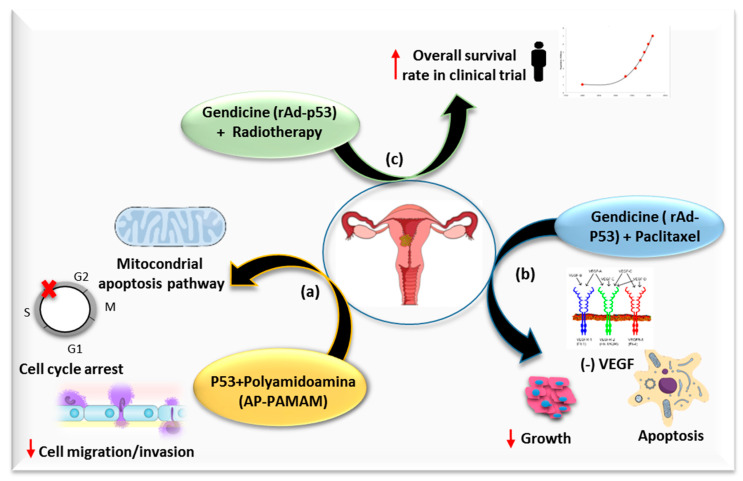
Schematic representation of some strategies focused on p53 restoration in cervical cancer developed to be used alone (**a**) or in combination with (**b**) chemotherapy or (**c**) RT.

One of the most studied genes in cancer is p53, a protein involved in cell cycle arrest, apoptosis, autophagy, inhibition of proliferation of tumor cells and chemo/radiosensitivity (Figure 2) [60]. This gene is inactivated by E6 protein in a high percentage of cervical cancers [61], so p53 based gene therapy has been widely studied in cervical cancer. Han et al. showed that p53 delivery by a polyamidoamine derivative (AP-PAMAM) achieved an antiproliferative effect by the activation of a mitochondrial-dependent apoptosis pathway and the induction of cell cycle arrest at S phase, in addition to suppressing the cell migration and invasion of cervical cancer cells [37]. A drug combination of recombinant adenovirus (rAd)-p53 and PTX enhances growth inhibition and apoptosis with respect to a single drug with a synergist effect. This effect may be related to the inhibition of VEGF expression, since the expression level of VEGF was lower in PTX, rAd-p53 and a combined group than in the control group. The inhibitory effect was more evident in rAd-p53 in combination with the PTX group than that of single drug group [38]. Furthermore, there are more promising data coming from the use of Gendicine, a gene therapy product approved for clinical use in China in 2003. This therapy is based on the injection of a recombinant human adenovirus engineered to express wildtype-p53 (Ad-p53), and has been used in patients with cervical cancer in combination with RT in a randomized control clinical trial. A total of 104 patients with locally advanced cervical cancer (FIGO stage IIB-IIB) were enrolled in the trial. Intratumoral injections with rAd-p53 and pelvic RT plus brachytherapy were administered to 69 patients (group PRT). The 5-year overall survival (OS) rate and 5-year PFS rate were higher than that of the RT control group (group RT), with 35 patients (OS rate-5 year: PRT 74.2% vs. RT OS 56.7%, *p* = 0.084; PFS rate-5 year PRT 7.7% vs. RT 59.6%, *p* = 0.047), without increasing the adverse events [39]. Although there are encouraging results in locoregional tumor control with Ad-p53 in combination with RT (the group PRT and the group RT developed 5-year locoregional recurrence rates of 6.2% and 28.6% respectively, with *p* = 0.003), it should be noted that there were no statistically significant differences in 5-year OS rate, PFS rate and distant metastases rate (21.3% vs. 25.85%, *p* = 0.662). Statistical significance could probably be achieved if the study size were increased. Furthermore, there was not a proper design of the control group, since the standard management for Stage IIB and IIIB is platinum-based CRT, not RT alone. Therefore, it is advisable to build an adequate control group for future clinical trials based on the use of Gendicine.

Another tumor suppressor gene studied in cervical cancer is retinoblastoma protein zinc finger gene (*RIZ*), specifically its RIZ1 expression products, which can induce cell cycle arrest and apoptosis. Cheng et al. found the upregulation of RIZ1 in SiHa(HPV16+ve) cells reduced cell proliferation, and treatment with PTX increased the expression level of RIZ1 and enhanced tumor suppressive function, indicating that RIZ1 combined with PTX has a synergic effect [40]. The same group also found that RIZ1 overexpression combined with RT achieved an increased apoptosis rate and DNA damage in HeLa and SiHa cells, with an enhanced radiosensitivity of cancer cells [41]. It would be interesting to study the effect obtained with the combination of CRT with upregulation of RIZ1, since the synergic effect obtained may be even greater, but always with a careful analysis of the possible increase in adverse effects.

Another strategy focuses on PEDF, a glycoprotein with antitumor properties and with low expression in cervical cancer. Yang et al. developed FLP to deliver a *PEDF* gene to cervical cancer cells, and found significant inhibition of the growth, and suppression of adhesion, invasion and cervical cancer cell migration in vitro. In addition, its intraperitoneal (ip) injection in an abdominal metastatic tumor model of cervical cancer showed tumor suppression by inhibiting neovascularization and cell proliferation and inducing apoptosis of tumor cells in vivo, with relatively non-toxic side effects [26].

Finally, protein tyrosine phosphatase receptor J (PTPRJ) is a tumor suppressor that is down regulated in the human cervical tumor tissues. Its overexpression showed a significant suppression of cell viability, growth and migration in the human cervical cancer cell line C33A cells, via suppression of the Janus kinase (JAK)1/ Signal transducer and activator of transcription (STAT)3 pathway and the downstream factors of STAT3, such as cyclin D, Bax, VEGF and matrix metalloproteinase (MMP)2. Additionally its knockdown increased the C33A cell resistance to apoptosis induced by 5-fluorouracil (5FU) [42]. However, this study presents some limitations that have to be resolved before considering it as a potential therapeutic strategy. Thus, the use of only the C33a cell line—which is negative for HPV DNA and RNA—as an experimental model is not enough and cannot be considered a representative study sample, taking into account the cellular heterogeneity of cervical cancer. Therefore, the results that might be obtained in other cell lines positive for HPV would be very interesting, in order to better understand the reality of cervical cancer. Moreover, preclinical assays are needed to validate the effectiveness of this system.

Although many tumor suppressor genes have been studied for targeted therapy in the field of oncology, in recent years the studies of p53, RIZ1, PEDF and PTPRJ stand out for the treatment of cervical cancer. We can see that the results at the preclinical level are encouraging. However, the only strategy that has reached treatment in humans is p53-targeted therapy, which has had poor results. In conclusion, different strategies have been described in the literature, whose objective is to re-establish the activity of various tumor suppressor genes. However, in our opinion, they should be combined with other therapeutic strategies in order to increase the efficacy and selectivity of the system against cervical tumor cells and avoid the risk of developing resistance mechanisms.

##### Blocking Oncogenic Expression

Oncogenes are a potential target for gene silencing therapy, through several strategies such as using antisense oligonucleotides (ASO) binding to the target mRNA to block its transduction, or use of RNA interferences (RNAi) mediated by small interfering RNAs (siRNAs) or short hairpin RNAs (shRNAs) [25].

The main tumorigenic effects of the HPV have been attributed to the expression of *E6* and *E7* oncogenes. Different gene therapy approaches have been directed to block their expression (ASOs, ribozymes, siRNAs) to restore the function of these tumor suppressor proteins, and have proven to be effective in several studies, showing significant biological effects on survival of human cancer cells in vitro and in vivo. Non-replicative rAd vectors expressing artificial miRNAs directed against *E6* achieved in vitro E6 knockdown in HeLa and SiHa cells, leading to cell death by apoptosis via the intrinsic pathway. In addition, HeLa tumors in nude mice received daily intratumoral injections of 5.10^9^ pfu of vector over 5 days, and the growth of tumor was significantly inhibited, increasing the OS rate [43]. This kind of vector has recently been used to simultaneously inhibit E6 and E7 by shRNA, both in vitro and in vivo [44]. However, the use of a non-replicative rAd has the limitations of not passing from transduced cells to the neighboring negative cells, which translates into poorer vector dissemination. In addition, intratumoral administration is a limited strategy in regional infiltration and metastasis. Reschener et al. studied a strategy of gene silencing activated under illumination, using an E6-ASO tethered to a photoreactive polyazaaromatic ruthenium (Ru^ll^) complex. This system was able to irreversibly crosslink E6 oncogene under visible illumination and inhibit the expression of the complementary targeted gene. Consequently, p53 degradation was prevented, resulting in the inactivation of SiHa cell proliferation in monolayer and in three-dimensional cultures [45]. This approach showed good results in vitro, but, due to the difficulty involved in achieving activation under illumination in living organisms, its possible application in vivo and in humans remains to be elucidated. On the other hand, E6 suppression by transfecting HPV16 E6 siRNA restored p53 functions and sensitized the SiHa cells to apoptosis by cisplatin. This apoptotic effect was enhanced when death ligands, such as recombinant human TNF related apoptosis-inducing ligand (TRAIL) or anti-Fas antibody, were added, activating both intrinsic and extrinsic apoptotic pathways [62]. This double approach to the apoptotic pathways hinders possible mechanisms of escape from the treatment, since the cancer cells must jump two different mechanisms of apoptotic inhibition. Other strategies focused on these oncogenes used their disruption through the genome editing technology Clustered Regularly Interspersed Short Palindromic Repeats (CRISPR)-caspase 9 (Cas9), a promising tool that allows the inactivation of oncogenes [46]. Alternatively, using transcription activator-like effector nucleases (TALEN) [47], which showed they lead to growth inhibition and apoptosis of HPV positive cervical cancer cell lines, is also being studied in a phase I clinical trial for the treatment of CIN-1 patients. These patients have documented HPV16 or HPV18 infection, are married, have no fertility requirements and have had no administration of hormones in the last six months (NCT03057912). The expression of HPV16 E6/E7 is controlled by a common promoter, siRNA. Targeting this promoter was effective in knocking down E6 and E7 expression, and consequently induced decreased proliferation and increased apoptosis of cervical cancer cells in vitro and in a xenograft mouse model. This was perhaps due to the increased expression of p53 protein and decreased expression of p16 protein [48]. It is a strategy based on epigenetics, a widely expanding field, and relies on changes in gene expression without altering the DNA sequences, in this case through regulation of oncogene promoters. However, the moderate RNAi efficiency achieved in this study leads us to think that it would also be useful to focus on improving those factors that contribute to the efficiency of RNAi, such as finding better carriers. Finally, the combination of siRNA against the oncoprotein E7 and the antiapoptotic myeloid cell leukemin (MCL)-1 protein showed a decrease in the viability and induced apoptosis of HPV16 and HPV18 cell lines, without adverse effects on surrounding healthy tissue. In addition, its delivery by PEG-lipoplexes induced an efficient mRNA knockdown in vitro and after vaginal administration achieved complete coverage of the mucosal epithelium on mice [49]. Moreover, estrogen imbalance is an important factor in the development of cervical cancer. An adenovirus expressing a dominant-negative estrogen receptor gene (Ad-ER-DN) to block estrogen receptors, has been used in CaSki cervical cancer cells, achieving reduction in the expression of *E6* and *E7* oncogenes, blocking cell proliferation and inducing apoptosis [50]. However, these effects were observed only in approximately 15% of the cancer cells, a considerably low percentage. This fact demonstrates the need to combine different therapeutic mechanisms to achieve more desirable efficacy. In a similar vein, the same research group studied the use of Ad-ER-DN combined with cisplatin and PTX, achieving synergistic effects with enhanced cytotoxicity in the same cell line [51].

Furthermore, suppressing the expression of other genes such as an X-linked inhibitor of apoptosis protein (*XIAP*), *MMP*, Asparaginase like 1 (*ASRGL1*) and human telomerase reverse transcriptase (*hTERT*) are also under investigation and there have been promising results. In this context, XIAP is another anti-apoptosis protein that is important in maintaining the cellular immortality of cervical cancer cells. Its suppression by siRNA delivery showed a cleaved caspase-3 activation and subsequent cell apoptosis in tumor tissue, resulting in a good therapeutic effect on human cervical cancer xenograft mice [52]. In addition, MMP degrades extracellular matrix components, playing a key role in the differentiation and motility of cells. MMP-2 and MMP-9 are involved in metastasis due to their ability to degrade collagen type IV of the basement membrane of blood vessels, and their overexpression is related to the progression of cervical carcinoma. Silencing *MMP-9* gene expression by oligonucleotide shRNA in HeLa cells showed a reduction in the speed of cell migration [53]. Pentraxin 3 (PTX3), a molecule involved in MMP expression, is a component of innate immunity whose knockdown inhibits MMP-2 and MMP-9, decreasing migration and invasion of cervical cancer cells in vitro and in vivo [54]. The target therapy against MMP allows us to act against the main prognostic factor of cervical cancer, its lymphatic and metastatic extension. Nevertheless, it does not act against the proliferation of primary tumor, which makes it necessary to combine it with other therapies that do, and the development of studies in this direction is interesting.

ASRGL1 is an enzyme overexpressed in cervical cancer. Xiao-Feng et al. used an ASRGL1-shRNA-expressing lentivirus to study the effect of downregulation of this enzyme in SiHa cells [55]. A significant inhibition of cell multiplication was found, in relation to reduced expression of cyclin dependent kinases (CDK)2 and cyclin A2, and in addition enhanced cellular apoptosis with an increased expression of Bax and a decrease in Bcl-2. These results indicated that ASRGL1 could regulate the cell cycle and act as an anti-apoptotic factor. *hWAPL* is also a gene that plays a key role during the development of cervical cancer, and the significant decrease in its mRNA levels achieved by Zhang et al. led to a decreased rate of proliferation of HeLa cells [34]. Finally, hTERTs confer immortality on cancer cells, and are significantly upregulated in cervical cancer. Several studies have demonstrated that the knockdown of hTERT via siRNA effectively inhibited the expression of telomerase activity in cervical cancer cells leading to apoptosis both in vitro and in vivo [56,57]. Another way to get telomere shortening is through knockdown of HMBOX1, which has been shown to increase the radiosensitivity of HeLa and C33A cells [58]. This is a strategy that has not yet been studied in vivo, and therefore it is important to study if an increase in radiosensitivity achieved in these conditions could be transferred to the clinic.

Other studies focused on targeting of upstream kinases. In this context, the inhibition of protein phosphatase 2A (PP2A) activates p53, by phosphorylation at Serine20 and Serine46 residues mediated by CDK-5, restoring p53 functionality and resulting in a regression of tumor growth in vivo [63]. Moreover, Matsushita et al. used a therapeutic strategy to suppress c-myc, an important factor in cell proliferation and tumorigenesis, based on a c-myc transcriptional repressor (FIR). They used a fusion gene-deficient Sendai virus (SeV) as a vector, which is not pathogenic in humans and does not affect chromosome DNA in host cells. This shows a high gene transduction efficiency with significant antitumor effects and apoptosis induction due to the decrease in c-Myc in HeLa cells. Furthermore, it showed strong suppression of tumor growth with no significant side effects in vivo [59]. Although the authors indicate that there were no significant side effects, there is no detailed analysis of this aspect in the results section. Moreover, taking into account the ability of the SeV vector to transfer genes efficiently to a wide spectrum of cells, we consider that more data in this regard are necessary before future clinical application is considered.

In conclusion, several works have studied the treatment of cervical cancer by blocking the expression of different oncogenes. Many strategies have been studied, focusing on genes already studied in other cancers such as XIAP, hTERT, MMP or PP2A. However, due to the importance of the HPV-induced oncogenic process, the main efforts have been directed at E6 and E7 oncoproteins, with different silencing strategies. These strategies include: recombinant Ad vectors expressing artificial miRNAs, gene silencing activated under illumination, their disruption through the genome editing technology, and even epigenetic strategies targeting an HPV E6 / E7 common promoter. Among these, E6 and E7 are probably ideal targets for the development of novel therapeutic strategies. They represent proteins that are only present in HPV-infected cells and are therefore therapies that would not affect healthy tissue, achieving high selectivity.

##### microRNAs

**Figure 3:**
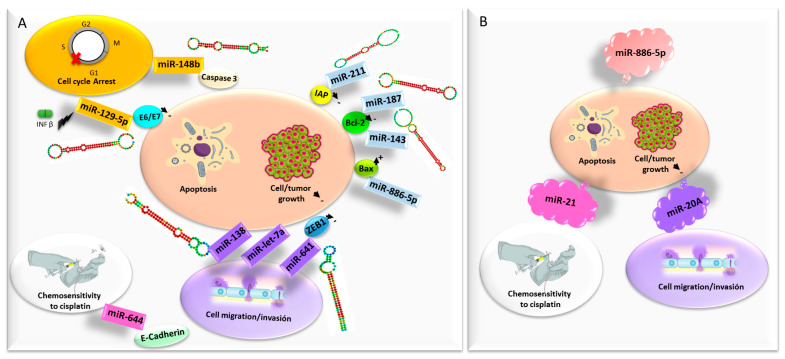
miRNAs as a therapeutic target against cervical cancer. Some miRNAs act as a tumor suppressor since they regulate oncogenes and are underexpressed in cervical cancer (**A**) while other miRNAs have an oncogenic capacity and are upregulated in cervical cancer cells (**B**). This feature makes them an attractive target for the treatment of cervical malignancies. miRNA images were obtained from Wikipedia.

The deregulated expression of non-coding RNAs of small size, microRNAs (miRNA), has been reported in the initiation and promotion processes of cervical cancer development. This means that miRNAs, with multiple biological functions implicated in cervical carcinogenesis and progression, show promise as novel therapeutic targets for the treatment of cervical malignancies. This is reflected in a recent in-depth review by Tornesello and collaborators [64]. Some miRNAs act as a tumor suppressor since they regulate oncogenes and are underexpressed in cervical cancer (Figure 3). In this context, in vitro lipofection of miR-187 into SiHa cells significantly reduced cell growth and induced apoptosis via downregulation of Bcl-2 [65] and inhibited the growth in nude mice [66]. Another important tumor suppressor of miRNA in cervical cancer is miR-143, which regulates several genes including *k-Ras*, *Macc1*, and *Bcl-2*. Liu et al. demonstrated that overexpression of miR-143 in HeLa cells resulted in suppression of Bcl-2, with inhibition of HeLa cell proliferation and the promotion of apoptosis [67]. In addition, Mou et al. have reported that overexpression of miR-148b induces G1/S-phase cell cycle arrest and apoptosis in a caspase-3-dependent manner in HeLa cells [68]. miRNA 211 plays a regulatory role in cervical cancer cells, and its transfection into SiHa cell lines inhibited the growth and promoted apoptosis of cancer cells via downregulation of inhibitor of apoptosis proteins (IAP) [69]. There are, therefore, different miRNAs that act at different levels in the apoptosis pathways that promote them, thus achieving a potential antitumor effect. Overexpression of miR-138 in cervical cancer cells suppressed cell migration and increased apoptosis in vitro, and inhibited tumor growth of cervical cancer models in vivo [70]. Furthermore, miR-641 expression is decreased in cervical cancer tissues and is correlated with the FIGO stage. Upregulation of miR-641 inhibits cervical cancer cell proliferation, migration and invasion, and promotes cell apoptosis, through downregulation of zinc finger e-box binding homeobox (ZEB)1 [71]. It is a miRNA that not only has therapeutic potential but also has prognostic value. miR-664 upregulation inhibits cervical cancer cell migration and increases chemosensitivity to cisplatin, with the possible participation of E-Cadherin [72]. miR-let-7a expression is decreased in SiHa and Hela cells, and its ectopic expression inhibited cell proliferation, migration and invasion in cervical cancer cells, facilitating apoptosis. In addition, it inhibited tumor growth in the mice xenograft model. Among others, miR-let-7a downregulates the expression of pyruvate kinase muscle isozyme M2 (PKM2) [73]. miRNAs can be induced by interferon (IFN)-β in cervical cancer cells, and these miRNAs can mediate E6 and E7 expression. Specifically, Zhang et al. found that miR-129-5p overexpression induced by IFN-β downregulated HPV18 E6 and E7 expression. Exogenous miR-129-5p inhibited cell proliferation, increased the arrest at the G0-G1 phase and promoted apoptosis of HeLa cells [74]. This work offers an interesting method with which researchers could study how different conventional treatments of cervical cancer (for example cisplatin) can modify the expression profile of different miRNAs and exert part of their action by modulating them. The use of these miRNAs by exogenous introduction into the cancer cells would allow us to achieve this therapeutic effect, perhaps avoiding some of the adverse effects of conventional therapy that may be due to other molecular mechanisms. 

Some miRNAs also have an oncogenic capacity (Figure 3). miR-21 is considered to be the most well-known oncogenic miRNA, and tumor suppressor gene phosphatase and tensin homologue deleted on chromosome ten (*PTEN*) is one of its various target genes. The use of siRNAs to silence miR-21 expression in cervical cancer cell lines showed increased locoregional *PTEN* gene expression, inhibition of cell proliferation, and led to cell death by autophagy and apoptosis, mediated by caspase-3/7 [75]. In another previous study, miR-21 was inhibited by TALEN in HeLa cells, also achieving increased levels of PTEN protein expression, as well as other genes related to cell environment interactions. Other results of this study obtained decreased expression of genes related to the redox regulation, whereas the sensitivity of cancer cells to cisplatin was enhanced [76]. Therefore, there may be other potential molecular pathways in cervical carcinogenesis that could be influenced by this silencing, representing a potential focal point of study for future work. Another miRNA overexpressed in cervical cancer is miR-886-5p, which negatively regulates the expression of the apoptotic protein Bax. Silencing miR-886-5p in SiHa cells increased levels of Bax and apoptosis [77]. Kang et al. found that miR-20a is upregulated in cervical cancer tissue. Inhibition of miR-20a in the cervical cancer cell lines HeLa and C33A suppressed cell proliferation, migration, and invasion [78]. It seems that this effect may be due in part to upregulation of Tankyrase 2 (TNKS2), a new member of the human telomere-associated poly (ADP-ribose) polymerase family. However, since cell viability does not seem to be affected, it seems likely that there could be other mechanisms which have yet to be studied.

In conclusion, the discovery of aberrantly expressed miRNAs in cervical cancer has elucidated novel molecular mechanisms of cervical cancer tumorigenesis and provided new opportunities to translate non-coding RNA research into clinical settings. Many studies underlined that miRNAs play an important role in growth, differentiation, apoptosis, migration, and resistance to conventional therapies in cervical cancer. miRNA regulates multiple cellular events, and the same miRNA can function as an oncomiR in a particular cancer and as a tumor suppressor gene in a different malignancy. Strategies to silence the expression of some miRNAs, such as miR-21 (considered to be one of the most important oncogenic miRNAs), miR-20a or miR-886-5p have been shown to be useful in suppressing cervical cancer cell proliferation, migration, and invasion in preclinical studies. In addition, inducing upregulation of other miRNAs such as miR-187, miR-143, miR-148b, miR-211, mir-138, miR-641, miR-664, miR-let-7a or miR-129-5p have realistic potential to be applied as a cervical cancer treatment. More studies, primarily based on high-throughput sequencing technologies, are needed to further reveal the complexity of the interplay between miRNAs and the deregulation of new actionable metabolic pathways for treatment of cervical cancer. It is a field of study greatly expanded in recent years, so far with promising results, but there are still many aspects yet to be discovered.

#### 2.2.2. Suicide Gene Therapy

Suicide gene therapy is a strategy in which death inducing transgenes, called suicide genes, are introduced into cancer cells. There are two types of suicide genes: genes that encode toxic molecules to the cell, and genes that encode for enzymes not found in mammals, that convert an inactive substance into toxic metabolites [79,80]. Different gene-directed enzyme prodrug systems such as cytosine deaminase (CD)/5-flurocytosine (5-FC), herpes simplex virus thymidine kinase (HSV-TK)/ganciclovir enzyme (GCV) [81], nitroreductase (NTR)/CB1954 [82], and uracil phosphoribosyltransferase (UPRT)/5FU [83] have been used as therapeutic weapons in cervix cancer. These systems focus on the conversion of a non-toxic prodrug into a highly toxic metabolite, and are able to induce cell death. Indeed, Kim et al. used stem cells expressing a bacterial *CD* gene and/or an *IFN-β* gene, and showed decreased cell viability of HeLa cells in response to 5-FC. The anticancer activity was significantly increased when IFN-β was added by synergistic anti-cancer effects [27]. Hao et al. studied the combination of two suicide gene systems, CD/5-FC and HSV-TK/GCV, delivered by lentiviral vector microbubbles and ultrasonic radiation. They found complementary and synergic effects, and achieved significant inhibition of the proliferation and enhancement in apoptosis of HeLa cells [81]. Although they are good results, studies in animal models need to be done to analyze the possible adverse effects derived from this combination of suicide genes. In addition, we need to analyze how the results on proliferation and apoptosis can be modified by attenuating the ultrasonic radiation that reaches the cancer cells. Another suicide gene therapy studied in cervical cancer is the use of UPRT for efficient conversion of the prodrug 5FU into more toxic metabolites. In particular, Narayanan et al. used plant UPRTs from *Arabidopsis thaliana* instead of the bacterial one in HeLa cells. After treatment with 5FU, HeLa transfected cells showed induction of apoptosis and arrest in the S-phase by upregulation of cyclin D1 and p21, with a lesser survival efficiency than the control [83]. However, there is a potential bias due to selective reporting, since inferential statistics are made in cell viability but not in the assessment of apoptosis. In addition, the mechanisms underlying cell cycle arrest should be investigated, since the role of cyclin D1 as an oncogene that promotes cell proliferation is widely accepted.

To try to solve the problem of the bioavailability of the prodrug, new strategies have been designed in the field of suicide gene therapy to directly eliminate the tumor. In this context, Preston et al. developed a strategy based on a prokaryotic toxin-antitoxin (TA) system for the selective destruction of cervical cancer cells. The Kid-Kis TA protein pair functions as a rescue system in prokaryotic plasmid R1, Kid induces apoptosis and inhibits cell division while Kis neutralizes this effect. In this work a synthetic system, capable of activating in the presence of oncoprotein E6 in human cells, was developed in SiHa and HeLa cancer cells, achieving proliferative inhibition in HPV positive cells by induced apoptosis by Kid and healthy cell protection by Kis. This system is very interesting since it facilitates the protection of healthy cells and could be adapted to other oncogenes and toxins [84]. Furthermore, several proapoptotic genes have been successfully used to directly eliminate cervical tumor cells. In this way, TRAIL is a protein that causes apoptosis through the extrinsic pathway. Nasha et al. compared this suicide-gene therapy with PTX and irinotecan, in an HeLa ip tumor model, showing longer survival with reduced adverse effects [85]. Zheng et al. developed a nanoparticle to co-deliver TRAIL and endostatin, resulting in synergistic antitumor effects. The TRAIL/endostatin-loaded nanoparticles markedly increased cytotoxicity of the HeLa cells in comparison to control groups, and in a xenograft cancer model clearly decreased the tumor size [86]. These nanoparticles could efficiently deliver TRAIL and endostatin in combination with chemotherapy drugs, achieving a probable increased antitumor effect with lower adverse effects than the administration of chemotherapy by traditional routes. In addition, spermidine is a natural polyamine with many biological functions and was found to reduce the proliferation in a dose-dependent manner. It arrested the cell cycle at the S phase, and promoted both apoptosis and autophagy in cervical cancer cells [87]. Moreover, human lactoferrin (hLF) is a glycoprotein considered to be an important component of the nonspecific immune system that can reduce solid tumor growth. Li et al. described that the use of an rAd encoding hLF in Hela cells induces tumor growth inhibition due to cell cycle arrest in the G2/M phase. In addition, it elevated expression of Fas, a death-inducing receptor, and Bax, and decreased expression of the anti-apoptotic Bcl-2. Furthermore, they found prolonged survival in cervical-cancer-bearing mice that had been treated in this way [88]. Finally, the VP3 protein (apoptin) of chicken anemia virus is another viral protein with the ability to induce apoptosis in a broad range of cancers. It also transformed cells without affecting normal cells. In a recent study, apoptin induced apoptosis in HeLa cells through a caspase-dependent intrinsic pathway, independent of p53 activation [89]. Apoptin is found in the nucleus of tumor cells, whereas it is found in the cytoplasm in normal cells. This fact allows it to selectively kill tumor cells and, therefore, makes it highly attractive as a potentially safe therapeutic tool for clinical use.

In recent years, the uses of fusion proteins expressing two genes with antitumor effects have been used to enhance their cytotoxic action. In this sense, Yang et al. used a kind of Staphylococcus superantigen, SEC3, a potent cytokine inducer of human lymphocyte, alongside the endostatin gene, a potent inhibitor of angiogenesis, to inhibit proliferation and migration of HeLa cells [90]. Moreover, a recombinant plasmid containing the genes for *melittin* and *IL-2* was constructed with the aim of improving these functions and overcoming their shortcomings. *Melittin* is a cytolytic antimicrobial peptide that is reported to have cytotoxicity in various types of cancer cells, but with the problem of locoregional action. IL-2 is a molecule with a key role in antitumor immunity mediated by cytotoxic T lymphocytes (CTL), but with toxicity and a short half-life. The overexpression of this fusion protein inhibited the proliferation and induced apoptosis of HeLa cells [91]. These types of approaches can allow the cancer cell to be attacked on different fronts, not only using toxic genes but also using other types of genes, as we mentioned in the previous sections that act on the different hallmarks of cancer.

The combined administration of gene therapy with RT may enhance antitumor effect. Chen et al. gave CGV and combined RT with Cobalt-60 (60Co) radiation to human cervical cancer cells with transferred HSV-TK gene in vitro, and they found there was a significant increase in tumor cell growth inhibition. It also illustrated an increase in the apoptosis rate in the combined treatment group compared to the simple treatment group, showing a synergistic effect. In addition, the combined treatment group result was superior to the simple treatment group in the in vivo nude mice model. These results show that HSV-TK could enhance sensitivity of radiation, and RT could enhance the effect of a suicide gene to inhibit tumors [92]. The same happens with the combination of a NTR/CB1954 suicide gene system based on the conversion of prodrug CB1954 into an alkylating agent by Escherichia coli NTR, leading to DNA crosslinks and the apoptosis of cancer cells- and γ-rays on HeLa cells [82]. These works show how suicide gene therapy may sensitize tumor cells to the effects of ionizing radiation with excellent results both in vitro and in vivo. Efforts should be focused on the development of methods that aim to use this strategy to enhance RT in clinical treatments. The combination of gene therapy with chemotherapy has been also studied. Zhang et al. assessed a strategy using cisplatin and hTERT promoter-mediated horseradish peroxidase (HRP)/indole-3-acetic acid (IAA), another suicide gene system. This induced cell cycle arrest at the S phase and apoptosis in HeLa cells, achieving a significant reduction in cell viability, thus indicating the synergistic effect of this combined therapy [93].

Gene-directed enzyme prodrug systems and the use of different molecules with antitumor effects have been effective in treating cervical cancer cells in experimental trials. Among the multiple approaches described in this section, the use of Kid-Kis TA protein is probably one of the most interesting. Its activation is based on the presence of a specific oncoprotein of cervical cancer cells, HPV-E6, allowing good protection of healthy cells. Furthermore, TRAIL, either alone or in combination with endostatin, and hLF have been taken one step further than in vitro studies and have now been studied in animal models. However, successful suicide gene therapies depend on safe and efficient delivery systems. In this way, new studies should focus on this aspect in order to subsequently apply this strategy to clinical trials.

#### 2.2.3. Oncolytic Virotherapy

Oncolytic virotherapy offers a promising strategy for selective cervical cancer treatment. This approach is based on the use of replicating viruses with the aim of attacking tumor cells without harming healthy cells. Viruses have an affinity towards different receptors overexpressed in cancer cells, compared to normal cells. After infecting cancer cells, viruses self-replicate and infected host cells lyse, releasing subviruses that can infect other cells. In addition, lysed cells release tumor-specific antigens that can be used by antigen presenting cells, inducing an adaptive immune response against non-infected tumor cells. Finally oncoviruses can be constructed by knocking out, inserting or transferring an exogenous gene to increase the oncolytic efficacy of the virus [94].

Several natural viruses or genetically modified virus strains and their proteins have been evaluated for their oncolytic effect. These include adenovirus, vaccinia virus, herpes simplex virus, Newcastle disease, reovirus, and vesicular stomatitis virus (VSV). In this context, VSV, an RNA-based virus characterized by its rapid replication, is a potential candidate for cervical cancer treatment. VSV has the ability to target cells with defects in IFN signaling, without affecting healthy cells. Cervical cancer cells are defective in their IFN response due to the fact that the HPV E6 oncoprotein can inhibit the cellular IFN response, facilitating its infection and consequent destruction. Le Boeuf et al. confirmed a differential VSV infection and cytotoxicity in tumor xenograft nude mouse models and explanted tumor tissues from patients with cervical cancer [95].

Moreover, a combination of oncolytic virus and therapeutic gene could augment the killing of infected tumor cells. Indeed, HPV E2 can negatively regulate the expression of *HPV E6* and *E7* oncogenes, leading to cell cycle arrest, and is also involved in the induction of apoptosis. Wang et al. developed a tumor-specific replication-competent oncolytic rAd, M5, expressing the *HPV16 E2* gene. This combination of the oncolytic ability with gene silencing showed potent antitumoral efficacy in vitro and in vivo, due to viral replication, apoptosis and growth suppression [96]. Another system based on the insertion of a secreted isoform of VEGF inhibitor (VEGI-251) into an oncolytic adenoviral system with E1B 55 kDa gene deletion (ZD55) was described [97]. VEGI-251 showed that a combined effect of antiangiogenesis and mitochondrial mediated apoptosis activity enhanced the oncolytic ability of ZD55 in HeLa cells, in addition to suppression of tumor growth in xenograft tumors. Although these results are promising, there are still many studies to be done to determine the side effects of ZD-55-VEGI-251 before human translation can be achieved. In another study, ZD55 and TRAIL were used in combination with the histone deacetylase inhibitor suberoylanilide hydroxamic acid (SAHA), achieving a synergistic effect to kill HeLa cells by inducing G2 growth arrest and apoptosis. Moreover, in a mouse xenograft model it inhibited tumor growth. This synergistic effect is associated with the inhibition of the nuclear factor (NF)-κB signaling pathway and the enhanced activation of the extrinsic apoptosis pathway [98].

The synergistic effect of oncolytic virotherapy combined with RT has also been demonstrated in other studies. Wang et al. showed that the combined treatment with radiation and M5 Ad expressing *HPV16 E2* gene [96] or M6 Ad with antisense HPV16 E6 E7 DNA [99] inhibits the expression of these oncogenes and induced apoptosis in cervical cancer cells both in vitro and in vivo. M6 Ad could be improved by the introduction of the HPV18 E6 E7 DNA antisense, thus also achieving an antitumor effect in HPV18-related cancer cells. In another study, radiation responsive genes were inserted into a p53-targeted Ad, the promoter of early growth response-1 (Egr-1) and the proapoptotic protein TRAIL. This system significantly increased cell apoptosis induced by RT in cervical cell, and reduced tumor growth and enhanced tumor survival in xenograft mice [100].

In this section we present several viruses with the ability to selectively infect and destroy cervical cancer cells (e.g., VSV), viruses carrying particles with anticancer activity, such as molecules that inhibit E6 or E7 oncoproteins. These also have radiation responsive genes inserted into oncovirus systems with the aim of enhancing the effect of RT treatment. In short, oncolytic viruses clearly present a promising strategy in the approach to cervical cancer, thanks to their ability to target cancer cells by taking advantage of defects in IFN signaling. They can be used as vectors for one or more therapeutic genes, as seen in previous sections, accomplishing greater antitumor effect, or to achieve a synergistic effect with conventional therapies such as RT.

#### 2.2.4. Antiangiogenic Strategies

An important feature of cancer is its angiogenic capacity, important for cancer metastasis, which represents an ideal target for gene therapy.

VEGF is one of the most important growth factors associated with tumor angiogenesis, in addition to invasion and metastasis, and is overexpressed in cervical cancer tissues. As a consequence, it has received wide attention in gene therapy research. VEGF-C is one of the regulators of angiogenesis, through reduction in miR-326 expression and augmented cortactin expression, which results in enhanced cervical cancer invasiveness. miR-326 mimics transfection on SiHa cells, reduces cortactin expression induced by VEGF-C and, consequently, cell invasion. Similar results were found with the silencing of cortactin expression by specific siRNAs [101]. It is important to know the mechanisms underlying angiogenesis because, as this study has shown, anti-tumor strategies can stop this process, not only in related growth factors, but also by acting at different levels of the various pathways that control it. The remarkable fact is that radiation can increase expression of VEGF. In this sense, Qi et al. studied the impact of shRNA-*VEGF* in cell apoptosis and sensitivity to radiation in nude mice, and found RT combined with *VEGF* shRNA inhibited the growth of cervical cancer SiHa xenografts in nude mice. This reduced the tumor cells and cell atypia and increased tumor radiosensitivity via the improvement of the hypoxic microenvironment, in comparison to individual treatment [102].

On the other hand, the function of neuroepithelial transforming gene 1 (Net1), a guanine nucleotide exchange factor involved in several biological processes, has also been studied in cervical carcinogenesis. It has been seen that Net1 is overexpressed in cervical cancer tissues, correlating with microvessel density and aggressive clinical behavior (lymph node metastasis, vascular invasion, etc.). *Net1* siRNA decreased the proliferation, migration and angiogenesis capacity of SiHa cells, achieving a reduction in tumor growth and microvessel density of cervical cancer in vivo, maybe through VEGF downregulation [103]. We believe that the mechanism by which Net acts on angiogenesis should be studied further since it may also modulate other angiogenic factors.

In summary, VEGF and Net1 have been the main targets for studies on gene therapy and antiangiogenesis in recent years. These approaches have shown not only a reduction in blood or lymphatic vessel formation and migration, but also an improvement in terms of sensitivity to other treatments such as RT. The works on gene therapy to treat angiogenesis try to act on one of the main prognostic factors of cancer: its lymphatic or distant dissemination. Hence the importance of the different strategies described in this section. New efforts should be directed towards a better understanding of the various actors involved in this complex process, in order to develop better targeted therapies with the help of gene therapy.

#### 2.2.5. Genetic Immunopotentiation Therapies

Immunotherapy is a type of treatment focusing on the use of a patient’s immune system to fight diseases such as cancer. Immunotherapy can be divided into two approaches according to the mechanism of action of the therapeutic agent used, as well as to the status of the patient’s immune system. These approaches are:

(i) Passive immunotherapy; consisting of ex-vivo activated cells or molecules that, once inside the body, compensate for impaired immune functions. This category includes the administration of elements of the immune system such as tumor-specific antibodies, recombinant cytokines or pre-activated effector immune cells.
(ii) Active immunotherapy; which focuses on stimulation of host immune response in vivo to generate an antitumor response using vaccines, immunostimulatory cytokine among others [104].

The objective of anticancer immunomodulatory gene therapy is to induce or augment the immune response against cancer cells through gene therapy strategies (such as cytokine mediated gene therapy or cell carriers expressing immunomodulatory genes), thus improving effectiveness and toxicity.

HPV transcription and replication can be restricted through host restriction factors. However, HPV gene products such as E6 or E7oncoproteins target these restriction factors and abrogate their anti-viral effects (E6 and E7 directly counteract IFN and NFκB signaling pathways, and E2 can downmodulate IFN and STING) [105]. Immunotherapy is a promising strategy for cervical cancers because the tumors have HPV E6 and E7 antigens, specific antigens expressed by HPV-associated cancers that are not present in healthy tissues. This has favored the development and study of different strategies based on activation of the specific immune response against HPV oncoproteins and the administration of cytokines to activate T cell mediated responses. One of the most promising therapies in this field is T cell receptor (TCR) engineering, which is an adoptive cell therapy consisting of the use of T cells isolated from patient blood or tumor tissues. This is a genetic modification by introducing a TCR sequence, via a lentivirus or retrovirus vector, that targets a specific tumor antigen which has an important function in tumor cells. These modified T cells are then expanded in vitro to obtain sufficient numbers and re-infusion back into the patient [106]. Jin et al. developed T cells that target E7 with a high avidity TCR, and achieved regression of HPV-16+ human cervical cancer tumors in vivo [107]. This strategy is being used in patients with metastatic cervical cancers in a clinical trial (NCT02858310), whose results could be of great interest.

IL-12 is a cytokine with a central role in innate and adaptive immune responses [108], it is one of the most promising cytokines used in work on cervical cancer therapies. Its delivery using an adenoviral vector achieved inhibition of tumor growth and enhanced survival in a murine tumor model [33]. The treatment with the *IL-12* gene in an experimental HPV16-positive mouse tumor model showed an antitumor effect promoting the cellular immune response via shift to a Th1-cytokine profile (activation of CD8+ T cells and Natural killer (NK) T cells that can eliminate tumor cells). This treatment was associated with the inhibition of tumor growth and an increase in mouse survival rates [109]. Before IL-12 administration, HPV+ tumor cells showed predominant expression of the immunosuppressive cytokines IL-10 and transforming growth factor (TGF)-β1, which could downregulate the immune response favoring tumor implantation. Moreover, levels of IL-4 were also elevated. These findings suggest a Th2 and Th3 profile. With *IL-12* gene delivery, the levels of these cytokines decreased. These effects are not permanent and depend on the continuous administration of the plasmid into the tumor tissue, but it has the advantage of having lower toxic side effects than administration of the IL-12 protein. This strategy could be very attractive as an adjuvant therapy, once a delivery mechanism by which a sustained and delayed release of the gene to reach a more permanent effect is developed (e.g., as in depot presentations, where drug formulation is designed to provide slow, constant, sustained and prolonged action, to achieve a more permanent effect).

Another cytokine that has been studied as a possible therapy in cervical cancer is CXC chemokine ligand 10 (CXCL10). Liposome-encapsulated plasmid-encoding *CXCL10* may inhibit the growth of cervical carcinoma by modulating the expression of E6 and E7 oncoproteins, induction of apoptosis and angiogenesis inhibition [110]. Moreover, this plasmid can arrest the cell cycle at the G1 phase, promoting the efficacy of RT, because the cells are least sensitive to radiation at the S phase [111]. Since this cytokine has the ability to stimulate the migration and adhesion of T and NK cells, and the in vivo results come from athymic mice, a greater antitumor effect can be expected in immunocompetent humans. It would be interesting to verify this fact through clinical trials. In addition, treatment combined with intravenous administration of liposome-encapsulated plasmid-encoding *CXCL10* and RT showed a significant increase in the inhibition of growth. It promoted microvessel density reduction, decrease in cell proliferation and upregulation of apoptosis in tumor cells compared to each treatment alone [112]. However, this work did not evaluate the harmful effects derived from this combination, meaning it will be necessary to study this aspect before its possible translation into humans.

Human IL-15 (hIL-15) is a cytokine with anti-tumor activities due to NK cell activation and immunoglobulin secretion, but it has a short half-life. Yiang et al. used a recombinant adenovirus-associate vector 2 (rAAV2) to deliver the *hIL15* gene into an HeLa cell, inhibiting cell growth in an in vivo model [113]. However, these results must be interpreted with caution since they were achieved in mice pretreated with rAAV2-hIL15 before tumor implantation. There were non-statistically significant results in mice treated after tumor implantation that might better represent what could happen in humans. Consequently, it would be advisable to modify this type of strategy to achieve efficacy in humans.

Another promising strategy in the field of immunotherapy against cervical cancer is the use of therapeutic DNA vaccines, since the E6 and E7 oncoproteins are optimal targets due to their tumor specificity. The aim of these therapeutic vaccines is to eliminate malignant lesions by generating T-cell mediated immunity against HPV-infected cells. Sun et al. developed a DNA vaccine encoding calreticulin (CRT) linked to E7. Intravaginal administration followed by electroporation was used to enhance the expression of the protein encoded by the delivered DNA plasmid. As a result of this, a potent E7-specific CD8 T-cell response was found, in addition to enhanced antitumor effects and survival of orthotopic TC-1 tumor-bearing mice [114]. The CRT/E7 DNA vaccine is being tested in clinical trials, about which we will talk in another section. It would also be interesting if the electroporation-enhanced vaginal administration was also transferred to the clinic. Recently, a DNA vaccine based on the union of the signal peptide (SP) of CRT, SA-4-1BBL (a form of the natural ligand for the 4-1BB co-stimulatory receptor of the TNF superfamily, with effects on innate, adaptive, and regulatory immunity) and *HPV-16 E7* oncogene was developed. This vaccine induced higher prophylactic and therapeutic effects in TC-1 tumor model in vivo compared to mice treated with SP-SA-4-1BLL or E7 only, in relation to an increase in E7-specific T cells producing IFN-γ. Furthermore, this vaccine established long-term immunologic memory that prevents recurrence [115]. The use of co-stimulatory molecules of the TNF superfamily seems to enhance the immune response achieved with vaccines. That fact alone would justify that any vaccine that is studied for the purpose of use in humans should take advantage of this feature and incorporate this strategy in preclinical trials. Another strategy to improve the vaccine efficacy is the use of immunoregulatory cytokines. In a recent study intravaginal immunization with pcDNA-3CRT/E7 co-administrated with DNA encoding IL-2 followed by electroporation in tumor-bearing mice, a stronger anti-tumor CTL response and enhanced antitumor effects were induced compared to mice treated with the vaccine alone [116]. This strategy is also being studied in clinical trials, as discussed below. The use of therapeutic HPV vaccines is being investigated not only in cervical cancer but also in pre-neoplastic lesions [117]. The aim is to achieve the clearance of infected cells through the induction of HPV-specific CD8+ T cells, producing local immune suppression in CIN lesions before cancer develops [118]. This may represent a more conservative strategy for the treatment of premalignant lesions, avoiding the effects derived from techniques such as cervical conization. This includes the excision of a portion of the cervix surrounding the endocervical canal, which can cause cervical insufficiency (cervical dilatation during pregnancy without contractions due to weakness of the cervix tissues, contributing to premature birth).

Furthermore, *light*, a TNF superfamily ligand is also used for immune system stimulation. Mayinuer et al. studied cervical cancer immunotherapy using a rAAV vector expressing LIGHT, also known as TNF superfamily member 14. rAAV-LIGHT showed a potent antitumor activity through augmentation of the recruitment of T cells into the tumor and the activation of antitumor immune responses, and tumor growth suppression in in vivo models [119].

As in other approaches, diverse combinatorial strategies have been tested to promote immunopotenting therapies. Cheng et al. studied the therapeutic action of a combination therapy based on dendritic cells transfected with the tumor-specific antigen E7 (DC-E7) and human sodium iodide symporter (*hNIS*) gene. This gene is a co-transporter of sodium and iodide ions whose expression in cancer cells allows the accumulation of therapeutic radioisotope as I-131. Using either single therapy with DC-E7 vaccine or I-131 induced retardation in tumor growth in a xenograft animal cancer model, with posterior regrowth. However, the combined therapy showed an important inhibition of tumor growth, causing the tumors in all mice to completely disappear [120]. In a recent study, NIS-based gene therapy has also been used under the control of the hTERT promoter. This strategy significantly reduced the survival of HeLa cells and inhibited the growth of a cervical cancer xenograft model by reduction in cell proliferation and increased apoptosis [121]. Therapeutic effectiveness depends on the dose and retention time of the radioisotope in the tumor expressing NIS. Therefore, future research should also focus on finding a radioisotope that reaches a higher level within the tumor cell and remains inside it for longer. In addition, the side effects that this technique can generate in those tissues which express endogenous NIS, such as the thyroid and stomach, should be studied. Another immunotherapy approach is the use of Cytokine-induced killer (CIK) cells, derived from peripheral blood mononuclear cells from different donors, which have antitumor activity against a variety of tumors. A recent work showed the possibility of improving CIK expansion and antitumor efficacy in vitro and in vivo through co-culture with dendritic cells with cytokine signaling 1 (SOCS1) expression suppressed. SOCS1 is an important factor in the control of adaptive immunity. This co-culture showed an increase in the secretion of cytokines such as IL-12 and IFN-γ, in addition to reducing the expansion time of CIKs [122].

In conclusion, genetic immunopotentiation is one of the most promising strategies in gene therapy for cervical cancer treatment. T cells have been genetically engineered to target E7 oncoprotein in human studies. Activation of the immune system with different cytokine genes, such as IL-12, IL15 or CXCL10, has been developed, and the use of IL-12 is being evaluated in clinical trials, as we will see later. The use of therapeutic DNA vaccines based on HPV proteins represents a promising approach in cervical oncological pathology due to their tumor specificity, leading to the recent development of multiple strategies based on these types of vaccines. The last evaluated strategy has been the genetic improvement of immune cells such as CIK cells. Many of these strategies have demonstrated such a degree of effectiveness that they have passed evaluation in humans, with multiple clinical trials focusing on immunopotentiation.

#### 2.2.6. Therapies Targeting Drug Resistance

The acquisition of chemoresistance is an important limitation in the treatment of cancer, since it entails a failure of the chemotherapy treatment. Different strategies of gene therapy can enhance the sensitivity of cervical cancer cells to chemotherapy. An example is the use of hTERTC27, a constructed hTERT C-terminal polypeptide reported to decrease cell proliferation by promoting chromosome end-to-end fusion achieving telomerase dysfunction. Lin et al. found that overexpression of hTERTC27 increased the sensitivity of HeLa cells to 5FU, inhibiting cell proliferation and promoting 5FU induced apoptosis by downregulation of Bcl-2 and upregulation of activated caspase-3 and -9 [123]. Since overexpression of this polypeptide also enhances the sensitivity of HeLa cells to hydrogen peroxide (H2O2) treatment, we can hypothesize that hTERTC27 overexpression could be sensitizing cervical tumor cells to other chemotherapeutics agents like PTX. Moreover, breast cancer (BRCA)1-interacting protein 1 (BRIP1) is a DNA-dependent ATPase and a DNA helicase that interacts with BRCA1 in its DNA damage repair functions, and single nucleotide polymorphisms in the *BRIP1* gene associated with the progression of cervical cancer. Lie et al. found that upregulation of *BRIP1* enhances the antitumor activity of cisplatin, promoting cell apoptosis and suppressing tumor angiogenesis, in HeLa cells. This enhanced chemosensitivity seems to be achieved by inhibiting Rac1 GTPase activation [124]. Furthermore, *NANOG* has a crucial role in stemness maintenance of CSCs, in addition to participating in chemoresistance and immunoresistance. Histone deacetylase 1 (HDAC1) transcription is induced by NANOG, which is key to these NANOG-dependent phenotypes of cancer cells. HDAC1 participates in the repression of cell cycle inhibitors CDKN2D and CDKN1B, and in the upregulation of MCL-1, an antiapoptotic molecule which confers immunoresistance and chemoresitstance. This fact causes HDAC1 inhibition, in combination with antigen-specific adoptive T cell therapy, to overtake immune refractory cancers, leading to immune-mediated tumor regression [125]. Strategies that block the NANOG-HDAC1 signaling pathway could resolve the problem of immune escape and chemoresistance and the stem-like state in cancer. Its use in combination with immunotherapy or chemotherapy could represent a promising future approach to cervical cancer. Another strategy to enhance the sensitivity of cervical cancer cells to cisplatin is the upregulation of miR-1284, which has low levels in cancer cell tissues and is an independent prognostic factor for cervical cancer. This promotion of chemosensitivity is made via suppression of High mobility group box 1 protein, encoded by the *HMGB1* gene, a nuclear protein that organizes the DNA and regulates transcription [126].

Since chemoresistance is a huge problem in cervical cancer patients, enhancing the sensitivity of cancer cells to chemotherapeutic agents has become crucial. As a result, the study of underlying molecular mechanisms of chemoresistance has attracted the attention of researchers. Furthermore, the development of different strategies that can overcome these barriers, such as the upregulation of genes involved in chemoresistance, like BRIP1, or the modification of the gene expression through epigenetics, represent an interesting field of study to try to apply to humans in the future.

## 3. Clinical Trials

**Figure 4:**
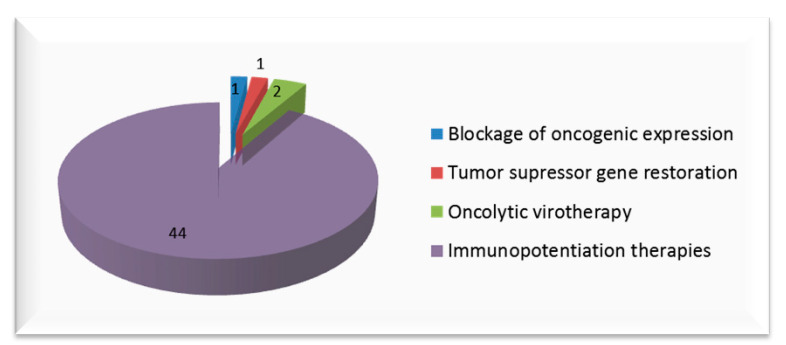
Number of ongoing or completed clinical trials in gene targeted therapy for cervical cancer worldwide from 1989 to 2018. They are grouped by the strategy used according to The Journal of Gene Medicine Clinical Trial site (source: http://www.abedia.com/wiley/indications.php) [127].

Clinical trials are an essential step in the validation of the different strategies previously mentioned in the approach to cervical cancer based on gene therapy. According to The Journal of Gene Medicine Clinical Trial website (source: http://www.abedia.com/wiley/indications.php) [127], there were a total of 2004 worldwide clinical trials based on cancer gene therapy from 1989 to 2018. These represented 66.6% of the total gene therapy clinical trials compiled in this registry. After an exhaustive study-to-study screening it was found that 47 of them were focused on cervical cancer (Table S1). Within these 47 studies, most evaluate different immunopotentiation strategies, mainly the use of vaccines to stimulate the formation of tumor-specific T cells, such as VGX-3100 or ADXS11-001. Two trials propose to study the efficacy and tolerability of oncolytic virus, and only one evaluates an oncofactor inhibition strategy, trial CN-0010 specifically analyses gendicine^®^ intratumoral injection combined with RT. There is also a lack of strategies for tumor suppressor gene restoration, which is only evaluated in trial CN-0059, studying TALEN and the CRISPR/Cas9 system (Figure 4).

To date, the clinical trial registry (Source: clinicaltrials.gov [128]) contains only nine clinical trials involving gene therapy strategies for cervical cancer, all of them focused on immunopotentiation (Table 2). The search criteria used are “gene therapy” and “cervix cancer”, with a subsequent one-to-one screening to discard those studies that did not really involve gene therapy strategies.

According to these two clinical trials registries, most current clinical trials concerning gene therapy in cervical cancer are based on immunopotentiation strategies. Therapeutic vaccines have been, and are currently being, explored in multiple clinical trials. Several therapeutic HPV vaccines have been evaluated in phase I clinical trials in patients with CIN2/3, utilizing different strategies to enhance their potency, achieving minimum toxicity and enhanced HPV specific response, but with limited clinical efficacy. It is necessary to explore new delivery strategies and incorporate more useful immune strategies to achieve a successful vaccine. These different strategies can provide a non-surgical option for the treatment of CIN2/3. A recent phase I clinical trial employed pNGVL4a-CRT/E7 (detox), a plasmid vaccine with coding sequences for HPV16 E7 linked to CRT, and examined its administration intradermal, intramuscular and intralesional. Although the vaccine was well-tolerated, histologic regression was observed in 33% of patients, a rate similar to that observed in unvaccinated patients [129]. A phase 2 trial used VGX-3100, a synthetic plasmid vaccine targeting HPV16 and HPV18 E6 and E7 proteins delivered intramuscularly, followed by electroporation with a CELLECTRA^®^ constant current device [130]. The histopathological regression and viral clearance were significantly greater in the therapeutic vaccination group compared to the placebo. This vaccine is being evaluated in a randomized, double-blind phase III trial (US-1528). A promising vaccine is modified vaccinia Ankara (MVA) E2, an attenuated virus derived from the vaccinia virus strain Ankara, containing HPV E2 oncoprotein, used to treat intraepithelial lesions associated with papillomavirus infection in a phase III trial. Its intralesional administration leads to the complete elimination of lesions in 89% of patients, and to the development of antibodies and a specific T cell response against papilloma infected cells in all treated patients. In addition this treatment induced eradication of HPV virus in 83% of total patients, inducing a strong immune memory [131].

HPV E6 and E7 vaccines are being evaluated not only in CIN but also in clinical trials of invasive cervical cancer. In Table 2 many clinical trials are summarized in which the safety and tolerability of these vaccines are evaluated. For example, safety and tolerability of VGX-3100 in cervical cancer is being assessed in a phase I trial (US-1283). Another phase I trial in The Netherlands (NL-0035) proposes to study a vaccine with E7 oncoprotein in combination with the fusion protein domain1 of tetanus toxin fragment C to enhance E7 specific T cell immunity. Trial US-1528 is an open-label study to analyze the efficacy of an HPV therapeutic vaccine containing a DNA plasmid targeting E6, E7 and IL-12 in combination with Durvalumab, an anti-PD-L1, in recurrent or metastatic HPV associated cancers. Another HPV vaccine under study is ADXS11-001, which contains live-attenuated Listeria monocytogenes encoding a nonhemolytic fragment of listeriolysin O protein fused to HPV16 E7. In a recent article the results of a phase II trial evaluating its administration with or without cisplatin in patients with recurrent or refractory cervical cancer have been published, showing a potential long-term clinical benefit and good tolerability [132]. A phase III study is ongoing to evaluate the efficacy of ADXS11-001 after CRT in locally advanced cervix cancer (US-1506).

Another growing strategy based on immunopotentiation is the use of T-cells, genetically engineered to express TCR- reactive, against tumor antigens. We can see different clinical trials in progress based in TCR receptors at clinicaltrials.org (NCT02280811, NCT02111850, NCT01583686, NCT02153905, and NCT02379520).

Outside immunopotentiation’s scope, there are very few clinical trials described in gene therapy for cervical cancer. Most of the strategies described above (e.g., virotherapy, suicide-based gene therapy, etc.) have not yet reached the phase of clinical trials. Recently, the results of a clinical trial focused on the use of the p53 gene in combination with chemotherapy were published. In this clinical trial, Xiao et al. evaluated the effect of intratumoral injection of rhAd-p53, a recombinant human adenovirus carrying the p53 gene, in combination with chemotherapy (cisplatin + vincristine + bleomycin) compared with chemotherapy alone. The trial comprised 40 patients with locally advanced cervical cancer (FIGO stages IB2-IIIA), who had not received prior chemotherapy or RT [133]. The efficacy of combined therapy was higher than in the chemotherapy group, with a significant shrinkage of the tumor size and a decrease in VEGF expression. There were no additional adverse events, except for self-limiting fever several hours after the injection, showing high efficacy and synergism. This allows the reduction in tumor staging in patients with advanced cervical cancer, providing conditions for surgical treatment.

## 4. Future Directions

Despite screening and treatment that have improved the prognosis of cervical cancer, many patients still suffer from recurrences and metastases, and their survival rates fail to improve. Therefore it is important to develop new treatment strategies to enhance patient prognosis.

Accumulating evidence suggests that CSCs may play an important role in cervical cancer tumorigenesis, tumor metastasis, cancer relapse and chemo/radio-resistance, thus representing promising targets to obtain a better therapeutic outcome. Cervical CSCs are a small subpopulation of tumor cells with self-renewing ability and can differentiate into heterogeneous tumor cells types, creating a progeny of cells that constitute the bulk of tumors. An important limitation is the difficulty of identifying cervical CSC, which has led to the search for different markers. These include ABCG2 (a drug efflux membrane transporter involved in multi-drug resistance), ALDH1 (a metabolic enzyme that acts as a stemness factor), CD133, CD49F, OCT4 (involved in the maintenance of stem cell pluripotency), Osteopontin (a matrix protein mediator of migration and metastasis), sex determining region Y(SRY)-box-2 (SOX2) and NANOG [134].

Different strategies have been evaluated to target cervical CSCs. On the one hand, the effect of miRNAs over CSCs has been evaluated. In fact, it has been shown that Mir-145 induces CSC differentiation and decreases cell invasion and colony formation, in addition to displaying a positive correlation to survival in cervical cancer patients. Indeed, injection of adenovirus carrying miR-145 in nude mice significantly reduced tumor growth, achieving increased survival [135]. In addition, introduction of miR-23b into cervical cancer cells altered stemness and cisplatin sensitivity [136].

On the other hand, certain substances have been shown to be effective in treating cervical CSCs, such as molecular iodine, that are able to inhibit proliferation and the ability to form tumors in mice by inhibiting CD49f, among other stemness markers [137]; Other substances include:

(a) Apigenin, a dietary flavonoid that inhibits the self-renewal capacity of HeLa CSCs by the inactivation of casein kinase 2α [138];
(b) Morusin, a natural compound isolated from the root bark of *Morus australis* with the ability to inhibit cervical CSCs growth and migration through NF-κB attenuation mediated apoptosis induction [139];
(c) Phenethyl isothiocyanate, a dietary constituent with cytotoxic activity through TRAIL-mediated apoptotic pathways [140];
(d) Doxycycline, a tetracycline used to treat a variety of infections that has the ability to inhibit proliferation and migration in HeLa-CSCs in addition to inducing apoptosis in vivo and in vitro [141];
(e) Zolendronic acid, that has been found to induce apoptosis and arrest cell cycle in CSCs, in addition to achieving attenuation of the stemness phenotype, leading to the inhibition of cervical CSCs proliferation in vitro and in vivo [142];
(f) A1E, a composition of 11 oriental medicinal plants that can inhibit CSCs and reduce the expression of stemness markers [143].

Hence, these substances could be used in combination with gene therapy to improve the response to conventional treatment.

Since HPV infection is involved in the majority of cervical cancers, immunotherapy is being widely studied. In this context, a promising approach is ex vivo adoptive T-cell therapy (ACT), with the goal of targeting and killing tumor cells, which has been studied in patients in a recent report by Stevanovic et al. [144]. Autologous tumor-infiltrating lymphocyte (TILs) from HPV-positive cancer, with reactivity against HPV E6 and E7, was administered in nine patients with metastatic cervical cancer, having previously undergone lymphocyte-depleting chemotherapy. Two patients experienced complete tumor responses, and one a partial response of 3 months in duration. Acute toxicities related to cell infusion or auto-immune adverse events were not reported, showing it to be a safe and tolerable treatment. In addition, HPV-specific T-cells persisted in peripheral blood for months after treatment [145]. In an ongoing phase II study (NCT03108495), which is currently recruiting patients, autologous TILs followed by IL-2 after a non-myeloablative lymphodepletion preparative regimen are being tested in patients with recurrent, metastatic or persistent cervical carcinoma. Another field of future research is chimeric antigen receptor (CAR)-T cells, genetically modified T cells that could be used to target the specific E6 and E7 oncoproteins. They have been developed against a wide variety of tumor antigens, mainly hematologic malignancies [114,115].

## 5. Conclusions

Despite advances made in the prevention of cervical cancer, either through immunization against different HPV serotypes using new vaccines or early detection through different screening methods, cervical cancer continues to represent a significant global health problem. No effective therapies exist for patients in an advanced stage. In this context, gene therapy is presented as a possible weapon that could be used to reduce morbidity and mortality.

Although development in the field of cervical cancer has been lower than in other types of cancers, multiple targeted gene delivery systems have been reported with encouraging preclinical results. HPV is omnipresent in the carcinogenesis process in the cervix, and this makes HPV E6 and 7 specific oncoproteins ideal candidates for the design of specific targeting gene delivery approaches to use against cervical tumor cells. Most clinical trials concerning gene delivery strategies in cervical cancer are based on immunopotentiation approaches, through the development of therapeutic vaccines or gene silencing strategies using gene therapy. However, the translation to humans has not yet shown a significant clinical benefit, due principally to the lack of efficient vectors. At present, real efforts are being made to develop new gene delivery systems, to improve tumor targeting and to minimize toxicity in normal tissues.

Finally, combining gene delivery strategies with RT/chemotherapy has been shown to improve the effectiveness and safety of the treatment. This suggests that the combination of targeted gene delivery systems with other emerging therapeutic strategies is a hopeful strategy—a good example being immunotherapy (adoptive T-cell therapy or novel monoclonal antibodies targeting immune check points such as pembrolizumab or nivolumab).

In conclusion, despite all the progress made, more work still has to be done and more clinical trials are needed to investigate current preclinical strategies and translate this promising tool into the clinic.

## Figures and Tables

**Table 1:** Most recent approaches focused on mutation compensation strategy for cervical cancer.

Strategy	Gene	Function in Cervical Cancer	Developed/Inhibition Strategy	Model	Reference
**Tumor suppressor gene restoration**	***p53***	Cell cycle arrest, apoptosis, autophagy, inhibition of proliferation of tumor cells and chemo/radiosensitivity	AP-PAMAM rAd-p53	In vitro *In vitro, in vivo,* in human	[37,38,39]
	***RIZ1***	Cell cycle arrest and apoptosis	pcDNA3.1(+)-RIZ1 plasmid	In vitro, in vivo	[40,41]
	***PEDF***	Antiangiogenic and antitumorigenic properties	FLP, ip administration	In vitro, in vivo	[26]
	***PTPRJ***	Regulate cell growth, differentiation and cell cycle	Lentivirus-pSicoR-PTPRJ	In vitro	[42]
**Blocking oncogenic expression**	***E6/E7***	Polyubiquination of p53, suppressing its function/pRb degradation, leading to S-phase entry, viral replication and maintenance	rAd-artificial miRNAs, intratumoral injection	In vitro, in vivo	[43,44]
			gene silencing activated under illumination	In vitro	[45]
			CRISPR-Cas9 TALEN	In vitro, in vivo, in human	[46,47]
			siRNA targeting E6/E7 promoter	In vitro, in vivo	[48]
			siRNA delivery by PEG-lipoplexes	In vitro, in vivo	[49]
			Ad-ER-DN	In vitro	[50,51]
	***XIAP***	Anti-apoptosis	siRNA	In vitro, in vivo	[52]
	***MMP***	Degrade extracellular matrix components, important in cell motility	shRNA Knockdown of PTX3	In vitro, in vivo	[53,54]
	***ASRGL1***	Cell cycle and anti-apoptotic factor regulation	shRNA-expressing lentivirus	In vitro	[55]
	***hTERT***	Lengthens telomeres in DNA strands, conferring immortality	siRNA Knockdown of HMBOX1	In vitro, in vivo	[56,57,58]
	***C-MYC***	Transcriptional factor involved in cell proliferation and tumorigenesis	Sendai virus carrying FIR	In vitro, in vivo	[59]

AP- PAMAM, 2-amino-6-chloropurine-modified polyamidoamine; rAd-p53, recombinant adenovirus p53, RIZ1, retinoblastoma-interacting zinc-finger protein 1; PEDF, pigment epithelium-derived factor; FLP, folate receptor α -targeted nano-liposomes; ip, intraperitoneal, PTPRJ, protein tyrosine phosphatase receptor J; pRb, retinoblastoma protein; miRNA, microRNA; CRISPR-Cas9, Clustered Regularly Interspersed Short Palindromic Repeats-caspase 9; TALEN, transcription activator-like effector nucleases, siRNA, small interfering RNAs; PEG, polyethylene glycol; Ad-ER-DN, adnovirus expressing a dominant-negative estrogen receptor; XIAP, X-linked inhibitor of apoptosis protein; MMP, matrix metalloproteinase; shRNA, short hairpin RNA; PTX3, pentraxin 3; ASRGL1, Asparaginase like 1; hTERT, human telomerase reverse transcriptase; HMBOX1, Homeobox containing 1; FIR, Far Up Stream Element-Binding Protein-Interacting Repressor.

**Table 2:** Current immunogene therapy clinical trials available in clinicaltrials.gov [128].

Therapeutic Strategy	Intervention	Reference	Phase	Year (First–Last Posted)
**Genetically engineered T-Cells and chemotherapy**	Fludarabine and Cyclophosphamide + E6 TCR (T-Cells genetically engineered to express T-Cell Receptors targeting HPV-16 E6) + Aldesleukin	NCT02280811	Phase 1 Phase 2	2014–2017
	Fludarabine and Cyclophosphamide + Anti-MAGE-A3-DP4 TCR (T-Cells genetically engineered to express T-Cell Receptors targeting the DP0401/0402 restricted MAGE-A3 tumor antigen) + Aldesleukin	NCT02111850	Phase 2	2014–2018
	Aldesleukin + Fludarabine and Cyclophosphamide + Anti-MAGE-A3 HLAA* 01-restricted TCR (T-Cells genetically engineered to express T-Cell Receptors targeting MAGE-A3 tumor antigen)	NCT02153905	Phase 1 Phase 2	2014–2018
	HPV Specific T Cells (modified genetically to be resistant to the TGF-beta) ± lymphodepletion (Cyclophosphamide and Fludarabine) and nivolumab	NCT02379520	Phase 1	2015–2018
**CAR transduced PBL and chemotherapy**	Fludarabine + Anti-mesothelin CAR transduced PBL (retroviral vector that contains a chimeric T cell receptor targeting mesothelin) + Cyclophosphamide + Aldesleukin	NCT01583686	Phase 1 Phase 2	2012–2018
**Vaccine**	pNGVL4a-CRT/E7(detox) vaccine, which targets HPV16 E7	NCT00988559	Not Applicable	2009–2016
**Vaccine and chemotherapy**	TA-HPV (HPVE6/E7 recombinant vaccine) and pNGVL4a-Sig/E7(detox)/HSP70 DNA vaccines ± imiquimod	NCT00788164	Phase 1	2008–2018
	Vigil (vaccine composed of autologous tumor cells which are transfected extracorporeally with a plasmid encoding for the gene for GM-CSF and a bifunctional shRNA that targets furin, a convertase responsible for activation of both TGβ1 and β2) + Atezolizumab	NCT03073525	Phase 2	2017–2018
	DNA plasmid-encoding IL-12/HPV DNA plasmid therapeutic vaccine INO-3112 (MEDI0457) in combination with durvalumab	NCT03439085	Phase 2	2018–2018

## Supplementary Materials

The following are available online at https://www.mdpi.com/2072-6694/12/5/1301/s1, Table S1: Gene therapy clinical trials for cervical cancer compiled in The Journal of Gene Medicine Clinical Trial website.
