# Supplementary material for: Targeted Gene Delivery Therapies for Cervical Cancer
Source: Cancers (Basel). 2020 May 21;12(5):1301. doi: 10.3390/cancers12051301 (PMC7281413; doi:10.3390/cancers12051301)
Supplement: Supplementary file 1 [file cancers-12-01301-s001.pdf]

cancers

# Targeted Gene Delivery Therapies for Cervical Cancer

Ángela Áyen, Yaiza Jiménez Martínez and Houria Boulaiz

**Table S1.** Gene therapy clinical trials worldwide on cervical cancer approved/initiated 1989-2018.

| Therapeutic Strategy | Intervention | Clinical Trial Reference | Phase | Date Approved |
|---|---|---|---|---|
| **Tumor suppressor gene restoration** | Intratumoral administration of Gendicina® + RT in Advanced cervical cancer | CN-0010 | Phase III | 2008-04 Open |
| **Oncofactor inhibition strategy** | Use of CRISPR-Cas9 and TALEN to produce disruption of HPV E6/E7 knockout, in CIN | CN-0059 | Phase I | 2017-07 Open |
| **Oncolytic virus** | Injection of rAd type 5 (H101), an E1b-deleted adenovirus, + RT and chemotherapy, in locally advanced cervical cancer | CN-0054 | Phase II | 2015-03 Open |
| **Oncolytic virus + immunopotentiation** | Intravenous and intramuscular administration of Oncolytic MG1 expressing HPV E6-E7 antigen + Adenovirus vaccine expressing HPV E6-E7 proteins + Atezolizumab, in HPV Associated Cancer | US-1760 | Phase I | 2018-05 Open |
| **Immunopotentiation** | Vaccinia virus encoding HPV E6 and E7 antigens in Cervical cancer | UK-0001 | Phase I/II | 1995-06 Closed |
| | Intramuscular administration of a vaccinia virus encoding an IL-2, in Stage I cervical cancer | CH-0035 | Phase I | 1999 Closed (2002) |
| | Intramuscular administration of a vaccinia virus encoding an IL-2, in Advanced cervical cancer | CH-0036 | Phase I | 1999 Closed (2002) |
| | Intramuscular administration of a vaccinia virus encoding HPV antigens and IL-2, in Stage I cervical cancer | XX-0006 | Phase I | 1999 Closed (2002) |
| | Intramuscular administration of a vaccinia virus encoding HPV antigens and IL-2, in Advanced cervical cancer | XX-0007 | Phase I | 1999 Closed (2002) |
| | ZYC101a, composed by HPV E6 and E7 antigens, in Ano-genital neoplasia 3 | UK-0071 | Phase II | 2001-01 Open |
| | Subcutaneous administration of RO5217790 (MVA-HPV-IL2, vaccine composed by a Modified Vaccinia Ankara (MVA) virus encoding HPV16 E6 and E7 antigens and an IL-2), in CIN 2/3 | BE-0024/ ES-0010 | Phase I | 2009-05-05 Open |
| | Intramuscular administration of RO5217790 (MVA-HPV-IL2), in CIN 2/3CIN 2/3 | US- 0958 | Phase II | 2008-12 Open |
| | Subcutaneous administration of TG4001 (MVA-HPV-IL2), in CIN 2/3 | FR-0032 | Phase II | 2004 Open |
| | Intramuscular administration of TG4001 (MVA-HPV-IL2), in CIN 3 | US-0307 | Phase I | 1999 Closed |

| | | | |
|---|---|---|---|
| Intramuscular administration of TG4001 (MVA-HPV-IL2), in Advanced cervical cancer | US-0309 | Phase I | 1999 Closed |
| MVA E2 recombinant vaccine in CIN 1/2 | MX-0001 | Phase II | N Open |
| TA-HPV, vaccine composed by HPV E6 and E7 antigens, in CIN 3 | UK-0041 | Phase I | 1996-05 Open |
| TA-HPV vaccine, in CIN 3 | UK-0042 | Phase I | 1997-08 Open |
| TA-HPV vaccine, in CIN 3 | UK-0046 | Phase I | 1996-09 Closed |
| TA-HPV, vaccine composed by HPV E6 and E7 antigens, in CIN 3 | UK-0047 | Phase I | 1998-01 Closed |
| TA-HPV vaccine, in High-grade ano-genital intraepithelial neoplasia | UK-0074 | Phase I | 2001-01 Open |
| GX-188E, a DNA-based Therapeutic Vaccine composed by HPV E6 and E7 antigens, administered Intramuscularly by Electroporation, in CIN 2/3 | EE-0001 | Phase II | N Open |
| GX-188E vaccine, administered Intramuscularly by Electroporation (EP), in CIN 3 | KR-0016 | Phase II | 2014-05 Open |
| Intramuscular administration of pNGVL4a-Sig/E7 (detox)/HSP70, vaccine composed by HPV16 E7 and heat shock protein 70, in CIN 2/3 | US-0595 | Phase I/II | 2003 Open |
| Intramuscular administration of pNGVL4a-CRT/E7(detox), DNA vaccine composed by HPV16 E7 and CRT, in CIN 2/3 | US-0984 | Phase I/II | 2009-06 Open |
| VGX-3100, a therapeutic DNA vaccine composed by HPV16 E6-E7 and HPV18 E6-E7 fusion proteins, administered intramuscularly by electroporation, in CIN 2/3 | US-0916 | Phase I | 2008-04 Open |
| VGX-3100 vaccine, administered intramuscularly by electroporation, in CIN 2/3 | US-1093 | Phase II | 2011-02 Open |
| VGX-3100 vaccine, administered intramuscularly by electroporation, in Cervical cancer | US-1040 | Phase I | 2010-04 Open |
| VGX-3100 vaccine, administered intramuscularly by electroporation + INO-9012, vaccine composed by IL-2, in Cervical cancer | US-1283 | Phase I | 2013-12 Open |
| VGX-3100 vaccine, administered intramuscularly by electroporation, in HSIL of cervix | US-1528 | Phase IV | 2016-04 Open |
| MEDI0457 (INO-3112), composed of HPV16 E6-E7 and HPV18 E6-E7 Fusion Protein + durvalumab (MEDI4736), anti-PD-L1, in Recurrent-Metastatic Human Papilloma Virus Associated Cancers | US-1686 | Phase II | 2017-10 Open |
| Vaccination with Listeria monocytogenes Expressing HPV16 E7, in Progressive, Recurrent and Advanced Cervical cancer | US-0592 | Phase I | 2003 Open |
| Intravenous administration of ADXS11-001, vaccine composed by HPV E7 | US-1082 | Phase II | 2010-12 Open |

| | | | |
|---|---|---|---|
| antigens, in Persistent or Recurrent cervical cancer | | | |
| Intravenous administration of ADXS11-001 vaccine, in Cervical cancer | US-1361 | Phase I/II | 2014-11 Open |
| Intravenous administration of ADXS11-001 vaccine ± durvalumab (MEDI4736), anti-PD-L1, in Recurrent-Metastatic Cervical or HPV+ Head and Neck Cancer | US-1362 | Phase I/II | 2014-11 Open |
| Intravenous administration of ADXS11-001 vaccine ± Epacadostat (INCB024360), in Stage I to IIIB Cervical Cancer | US-1430 | Phase II | 2015-05 Open |
| Intravenous administration of ADXS11-001 vaccine, following CRT as adjuvant, in High risk locally advanced cervical cancer | US-1506 | Phase III | 2016-02 Open |
| Intravenous administration of ADXS11-001 vaccine, following CRT as adjuvant, in High risk locally advanced cervical cancer | XX-0042 | Phase III | 2017-08 Open |
| Intradermal administration of TTFC-E7SH vaccine, composed of the fusion protein domain1 of tetanus toxin fragment C and the shuffled version of HPV16 E7, in Stage IV squamous cell carcinoma | NL-0035 | Phase I | 2013-04 Open |
| Intradermal vaccination with naked DNA, encoding the fusion protein of a carrier sequence sig-HELP-kdel and the shuffled version of HPV16 E6 and E7 antigens, in HPV induced (pre)malignancies | NL-0039 | Phase I | 2015-09 Open (2025) |
| Replication-incompetent Semliki Forest Virus replicon particles encoding HPV16 E6 and E7 antigens, in HPV-induced premalignant cervical lesions | NL-0030 | Phase I/II | 2011-04-07 Open |
| Intramuscular administration of a HPV16-Specific Therapeutic DNA-rVaccinia Vaccination + topical imiquimod, in CIN3 | US-0928 | Phase I | 2008-07 Open |
| CAR-T cells (GD2 CAR, PSMA CAR, Muc1 CAR, Mesothelin CAR) in Cervical cancer | CN-0105 | Phase I/II | 2017-11 Open |
| TCR gene therapy targeting HPV16 E6, in HPV-associated cancer | US-1331 | Phase II | 2014-07 Open |
| HPV-16/18 E6/E7- specific T-cells + Dominant Negative TGF-β Receptor II, in Relapsed HPV-Associated Cancers | US-1369 | Phase I | 2015-01 Open |
| HPV-16 E6 TCR ± - PD-1 Blockade Antibody, in HPV-Associated Cancer | US-1517 | Phase I/II | 2016-04 Open |
| HPV16 E7 TCR Engineered T Cells in Relapsed-Refractory HPV16+ Cancer | US-1793 | Phase I | 2018-07 Open |

Abbreviations: N = no data; Note: http://www.abedia.com/wiley/index.html, Database of clinical trials updated in December 2018, accessed March 10, 2020.
